# THREE-MONTH FUNCTIONAL TRAINING PROGRAMME IMPROVES KNEE JOINT FUNCTION IN ATHLETES POST-ACL RECONSTRUCTION SURGERY

**DOI:** 10.2340/jrm.v56.18701

**Published:** 2024-09-18

**Authors:** Chuanjia DU, Jiao JIAO, Jihe ZHOU, Bik Chu CHOW, Qiuqiong SHI, Xiaopei ZHANG, Siyu LIU, Jianchao YANG

**Affiliations:** 1 School of Sports Medicine and Health, Chengdu Sport University, Chengdu, China; 2 Department of Sport, Physical Education and Health, Hong Kong Baptist University, Hong Kong, China; 3 Dr. Stephen Hui Research Centre for Physical Recreation and Wellness, Hong Kong Baptist University, Hong Kong, China; 4 School of Fashion and Textiles, The Hong Kong Polytechnic University, Hong Kong, China; 5 Business School of Hohai University, Nanjing, Jiangsu, China

**Keywords:** anterior cruciate ligament, balance, stability, muscles activation, strength

## Abstract

**Objective:**

Rehabilitation and recovery duration following anterior cruciate ligament reconstructive surgery play a pivotal role in restoring optimal knee functionality in athletes. This study aimed to explore the impact of a 3-month functional training programme aligned with enhanced recovery after surgery on recuperation subsequent to anterior cruciate ligament reconstructive surgery.

**Design:**

A quasi-experimental study.

**Subjects:**

A cohort of 34 patients aged 14 to 24, who underwent anterior cruciate ligament reconstructive surgery and adhered to enhanced recovery after surgery protocols during the perioperative period, were allocated to an experimental group and a control group according to their eligibility, capacity, and willingness to engage in the functional training programme.

**Methods:**

The participants in the experimental group underwent a 3-month regimen of functional training following anterior cruciate ligament reconstructive surgery, whereas the control group followed a conventional recovery approach. Evaluations were conducted both prior to and following the 3-month recovery interval, utilizing the Y-Balance Test, Functional Movement Screening, and Isokinetic Knee Test.

**Results:**

Assessment outcomes of the Y-Balance Test, Isokinetic Knee Test, and Functional Movement Screening exhibited significant enhancement (*p* < 0.05) within the experimental group, as opposed to the control group. These findings underscore that those athletes who undertook the 3-month functional training regimen within the experimental group exhibited heightened dynamic balance capabilities, increased knee joint mobility, and enhanced stability compared with their counterparts in the control group.

**Conclusion:**

Consequently, this underscores the efficacy of the 3-month functional training protocol aligned with enhanced recovery after surgery, as a means to effectively facilitate recuperation subsequent to anterior cruciate ligament reconstructive surgery.

## LAY ABSTRACT

In this study, the effects of combining an enhanced recovery after surgery programme with a 3-month functional training regimen on knee joint function in athletes who had anterior cruciate ligament reconstruction surgery were examined. The findings revealed that athletes who underwent this combined intervention experienced significant enhancements in knee joint function, such as increased range of motion, improved muscular strength, enhanced joint position awareness, and better functional performance. The integration of the enhanced recovery after surgery programme with 3-month functional training can optimize the recovery process, enabling athletes to return to sports activities in a safer and faster manner. This approach shows promise in improving the quality of life and performance outcomes for athletes during their anterior cruciate ligament reconstructive surgery recovery.

Anterior cruciate ligament (ACL) injuries present a significant challenge in sports medicine, affecting over 200,000 athletes worldwide each year (1, 2). These injuries not only jeopardize an athlete’s career by limiting performance but also necessitate interventions such as anterior cruciate ligament reconstruction (ACLR) to restore stability and functionality to the knee joint (3, 4). ACLR, while effective, introduces a variety of rehabilitation challenges including altered neurodynamics such as changes in intracortical and corticospinal excitability, which can complicate the recovery process (5, 6). Complications such as inflammation, discomfort (7, 8), muscular atrophy (9), reduced joint flexibility (10), and decreased lower limb strength (11) further contribute to delays in returning to athletic activities.

The period immediately following surgery, particularly the first 3 months, is critical for successful rehabilitation (12). This phase requires carefully designed recovery protocols that aim not only to restore the physical attributes affected by the surgery but also to address the psychophysiological aspects of recovery. Unlike earlier rehabilitation strategies that primarily involved prolonged immobilization, contemporary approaches recommend a more dynamic (10, 13) and phased recovery process (11, 14). This includes early weight-bearing exercises that are designed to restore function while minimizing discomfort and enhancing joint mobility (1). Significant advancements have been made in rehabilitation strategies, such as the criteria-driven algorithm introduced by Myer et al. (15), which emphasizes structured, progressive programmes tailored to individual athletes. Such programmes have demonstrated efficacy in accelerating recovery, improving knee joint comfort, and facilitating a quicker return to competitive sports (16), Additionally, the Enhanced Recovery After Surgery (ERAS) protocols have emerged as a beneficial multimodal approach in surgical care, demonstrating significant improvements in postoperative knee functionality (17–22). Despite these advancements, the integration of ERAS with specific functional training post-ACLR remains underexplored.

This study is designed to address this gap by implementing a comprehensive 3-month functional training protocol, integrated with ERAS, aimed at enhancing the overall recovery process for individuals undergoing ACLR. By comparing the outcomes of athletes in the experimental group (EG), who will follow this integrated training regimen, with those in a control group (CG), we aim to demonstrate the superiority of our proposed approach. The primary hypothesis is that the EG will exhibit significant improvements in dynamic stability, knee strength, and overall athletic performance relative to their counterparts in the CG. This research seeks not only to validate the effectiveness of combining ERAS with targeted functional training but also to establish a new standard of care in the rehabilitation of ACL injuries.

## METHODS

### Participants and study design

This study employed a quasi-experimental design, which was approved by the University Research Ethics Committee (No: Cheng Ti Lun Li 2020-26). Participants were categorized based on their eligibility, capacity, and willingness to engage in the study’s specific conditions. More specifically, participants were assigned to the experimental or control group based on their capacity and willingness to engage in a “3-month regimen of functional training”. Those unable or unwilling to participate in the training due to economic, temporal, or regional factors were placed in the CG (23). The sample size estimation and power analysis were conducted to ensure the study had adequate power to detect differences, with an effect size of 0.97 (24), an alpha level of 0.05, a power of 0.8, and a rho value of 0.5. The resultant total sample size (*nn*), estimated to be 30 participants, was calculated through the use of G*Power software (https://www.psychologie.hhu.de/arbeitsgruppen/allgemeine-psychologie-und-arbeitspsychologie/gpower). This sample pool was subsequently bifurcated into 2 groups, each comprising 15 participants. Accounting for potential attrition, the study initially enlisted 45 athlete patients who had undergone ACLR at Sichuan Province Orthopaedic Hospital. Each patient provided informed consent and endorsed ERAS agreements, encompassing details concerning postoperative functional training procedures and potential factors influencing the trajectory of natural rehabilitation. Subsequent to collating demographic data encompassing variables such as age, gender, and the afflicted knee, the study proceeded with preoperative assessments. These assessments encompassed various evaluations, notably anterior drawer test, joint space tenderness, pivot shift, floating patella, knee range of extension and flexion, injured knee circumference, Beighton score, Tegner score, and Lachman tests. Preoperative magnetic resonance imaging (MRI) investigations facilitated the diagnosis of ACL rupture, unilateral ACL rupture devoid of contralateral knee injury, and other pertinent ligament injuries within the knee joint. The scope of injuries encompassed no ipsilateral meniscus injuries, with only partial debridement, or other specified conditions. It is pertinent to note that all patients underwent ACLR surgery performed by the same surgical team, wherein the reconstruction of the anterior cruciate ligament was executed through the utilization of autologous hamstring grafts. Notably, the perioperative period embraced the adoption of the ERAS protocol.

To ensure transparency and adherence to high research standards, our study followed the TREND (Transparent Reporting of Evaluations with Nonrandomized Designs) checklist. TREND is designed for studies like ours that do not use random participant allocation. It helps in clearly documenting the research process, including design, methods, results, and conclusions. This improves the reliability and clarity of the findings by ensuring all critical aspects of the study are thoroughly reported. Using the TREND checklist helps reduce biases typical in non-randomized studies and makes it easier for others to understand and replicate our work. This approach emphasizes our commitment to detailed and accountable research reporting.

### Experimental procedure

**Fig. 1:**
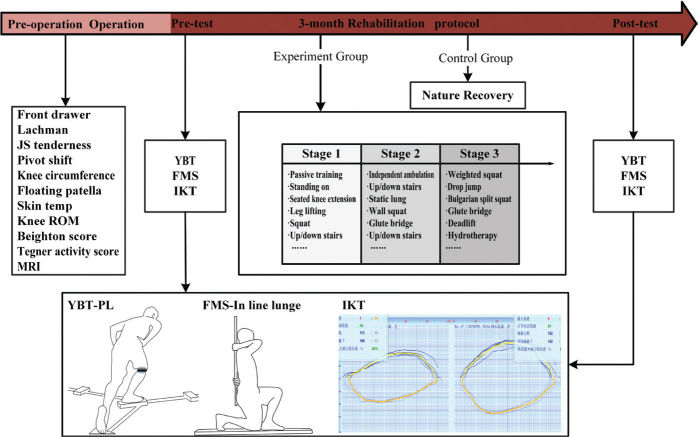
Flowchart of the experimental implementation.

The experimental procedure adhered to a predefined timeline (see Fig. 1). This timeline encompassed preoperative stages, intraoperative measures, an initial pre-test, a 3-month rehabilitation regimen for the EG, and a spontaneous rehabilitation approach for the CG. Both groups concluded the procedure with a subsequent post-test. Although patients in the CG did not receive the structured training programme used in the EG, they were informed about the content of the rehabilitation and encouraged to engage in rehabilitation training as much as their circumstances permitted.

### Preoperative assessments and measurements

Before surgery, several diagnostic and evaluative tests were conducted to assess each participant’s knee condition and readiness for surgery:

*Anterior drawer test, pivot shift test, and Lachman test.* These tests assess the integrity of the ACL.

*Joint space tenderness and floating patella tests.* Used to evaluate joint inflammation and patellar tracking.

*Knee range of extension and flexion.* Measures the degrees of movement possible at the knee joint.

*Beighton score.* Assesses overall joint hypermobility.

*Tegner activity level scale.* Quantifies activity levels to gauge pre-injury and post-surgery physical abilities.

Preoperative MRI was used to confirm ACL rupture and assess the state of other knee structures. All surgeries were performed by the same surgical team, utilizing autologous hamstring grafts within the framework of the ERAS protocol.

### ERAS nursing measures and surgical method

The preparatory measures undertaken prior to surgery, the surgical techniques employed, postoperative pain management methods, and early foundational rehabilitation care within the ERAS framework for a duration of 8–10 days were uniformly applied to both the EG and CG. Preoperative preparation encompassed the communication of the surgical plan, ERAS agreement, psychological readiness, and emotional equilibrium, as well as dietary and hydration arrangements. The arthroscopic ACLR surgery was conducted under femoral nerve block anaesthesia. Analgesia was achieved through the administration of parecoxib sodium and flurbiprofen axetil, aiming to mitigate pain and regulate inflammation.

### Functional training protocols

A 3-phase functional training protocol was methodically structured for the individuals within the EG, meticulously following a gradual progression principle as delineated in Table I. Specific objectives and the duration since surgery were pivotal factors influencing the advancement from one phase to the next. The inaugural stage, spanning 1–4 weeks, constituted the initial phase of foundational activity and movement function recuperation. Its temporal extent varied within this interval. The central aim during this stage lay in augmenting patients’ proprioceptive acuity and extending the range of motion (ROM) of the afflicted knee joint. Efforts were directed towards enhancing the muscular tension within the knee flexor and extensor muscle groups on the affected side. This was achieved through rudimentary training involving low to moderate resistance and a low-frequency regimen. Advancing to the second phase, which spanned from the 5th to the 8th week, the emphasis shifted towards a heightened pace of proprioceptive rehabilitation and fortification of the fundamental strength of the patients. In pursuit of this, weight-bearing resistance exercises were introduced to foster strength development and functional aptitude. The third phase, encompassing the interval from the 9th week to the 12th week, represented an essential phase of recuperation concerning dynamic neuromuscular strength, endurance, and equilibrium. The primary focus was to enhance balance proficiency. In this stage, more substantial training loads were incorporated to invigorate and enhance the patient’s core functional capabilities, thereby markedly refining both proprioceptive acumen and neuromuscular control proficiency. Conducting this comprehensive 3-month functional training regimen was a team of skilled rehabilitation therapists. Their responsibility extended to documenting the successful completion of each stage and assessing the patient’s position in terms of recovery progression. Furthermore, they diligently evaluated the efficacy of the training and adeptly adapted the programme in accordance with the evolving capabilities of the patients.

**Table I:** Enhanced Recovery After Surgery (ERAS) functional training protocol with three stages

Stage/goal	Training contents	Loading/sets
Stage 1 (1st–4th week) Proprioception enhancement and ROM expansion	Basic training: Passive knee extension and flexion Standing on both feet Standing on injured leg Seated knee extension Standing hip flexion to knee extension Leg lifting Movement up and down a set of stairs Ankle pumps with Theraband Squat Isometric quadriceps training Stimulate quadriceps by the neuromuscular low-frequency electrical stimulator, duration and frequency depend on specific circumstances	10–30 times*3 sets 3–5 min*3 sets 2–3 min*3 sets 10–15 times*3 sets 10–15 times*3 sets 10–15 times*3 sets 36 steps to and fro*3sets 20 times*3 sets 10–15 times*3 sets Maintain 10 s, 20 times*3 sets ---
	Relaxation activities: Stretch hamstrings and quadriceps Simulate injured knee by the ultrasound device Treat injured knee with the percutaneous electrical nerve stimulation device Relax injured knee’s muscles by the air wave pressure therapy device and the low-frequency electromagnetic pulse therapy device Ice compress	
Stage 2 (5th–8th week) Proprioception recovery and strength enhancement	Basic training: Independent ambulation Movement up and down a set of stairs	10 min*3 sets 36 steps to and fro*3 sets
	Strength training: Static lunge (keep thigh horizontal) Squat without load Wall squat Glute bridge Gymball squat Knee on one leg Gymball single-leg rollouts Glute bridge and knee extension	1–2 min*3 sets 20 times*3 sets 10–15 times*3 sets 1–2 min*3 sets 10–15 times*3 sets 10–15 times*3 sets 10–15 times*3 sets 10–15 times*3 sets
	Relaxation activities: Stretch hamstrings, quadriceps, and gluteus Foam roller relaxation Technique massage to relax the posterolateral and lateral iliotibial bundles of the knee Relax injured knee’s muscles by the air wave pressure therapy device	
Stage 3 (9th–12th week) Recovery of dynamic neuromuscular strength, endurance and balance	Basic training: Independent ambulation Jogging Bicycle	20 min*2sets 10 min*2sets 10 min*2sets
	Strength training: Lunge (forward and side) Jump deep Rear foot elevated split squat Bulgarian lunge squat Vaulting box Squat with load (keep thigh horizontal) Glute bridge Glute bridge and knee extension Hard pull on single leg Single leg bridge on box Hydrotherapy	20 times*4 sets 20 times*4 sets 20 times*4 sets 10 times*4 sets 20 times*4 sets 10 times*4 sets 20 times*2 sets 20 times*2 sets 10 times*4 sets 20 times*4 sets ---
	Relaxation activities: Stretch hamstrings, quadriceps, and gluteus (Static stretching and stretching with proprioceptive neuromuscular facilitation [PNF]) Foam roller relaxation Technique massage to relax the posterolateral and lateral iliotibial bundles of the knee Relax by fascia gun	

### Outcome measures

Outcome measures for both pre-test and post-test assessments included the Y-Balance Test (YBT), Functional Movement Screen (FMS), and Isokinetic Knee Test (IKT). Among them, IKT was the primary outcome. These assessments were designed to evaluate dynamic balance, functional movement, and knee strength respectively. The YBT and FMS were conducted around 10–12 days post-operation, while the IKT was performed approximately 30 days after surgery. Post-tests, inclusive of the YBT, FMS, and IKT, were carried out approximately 100 days following surgery. To prevent bias, evaluators were assigned to either the EG or CG in a blinded manner.

*Y-balance test (YBT).* Employed to gauge dynamic balance ability and neuromuscular control of the motor body (25, 26), the YBT involved measuring the distance from the anterior superior iliac spine to the lower edge of the medial malleolus for determination of lower limb length. In this test, the patient stood barefoot with their uninjured limb at the central point. Using the toe of the injured limb, the patient was instructed to push a cursor in 3 directions: anterior (A), posteromedial (PM), and posterolateral (PL). While sustaining balance, the patient aimed to push the cursor as far as possible in each direction. This process was repeated thrice for each direction, with the measurement taken as the most proximal reach indicator from the apex. The test was then repeated with the healthy limb. The composite score was calculated as an average of the 3 tests for each direction, normalized to limb length using the following formula (Eq. 1) (27):


Composite score = (A + PM + PL)/(3*LL)*100% Eq. 1

Here, A represents the reach distance in the anterior direction, PM in the posteromedial direction, PL in the posterolateral direction, and LL as lower limb length.

*Functional movement screen (FMS).* Developed by Gray Cook, FMS evaluates motive function through fundamental functional exercises, assessing flexibility, stability, and balance (28, 29). It is instrumental in identifying functional limitations, muscle imbalances, and asymmetries (30, 31). In this study, FMS gauged the influence of functional training on patients’ functional movements, thereby signifying the efficacy of the training. The evaluation involved patients performing a sequence of movements: deep squat, hurdle step, in-line lunge, shoulder mobility, active straight leg raise, trunk stability push up, and rotary stability. Subitems in FMS were scored on a scale of 0 to 3, where 3 indicated proficient task performance, 2 denoted task execution with compensation, 1 indicated inability to perform the task, and 0 implied pain during the task irrespective of quality. With a highest possible score of 21 (7 subitems) and a lowest of 0, scores below 14 indicated heightened vulnerability to injury, while scores above suggested reduced susceptibility to injury (32).

*Isokinetic knee test (IKT).* To assess knee joint strength and ROM, the Prima Plus isokinetic dynamometer (Easytech s.r.l., Borgo San Lorenzo, Italy) was employed. This apparatus facilitated the calculation of peak flexor torque and the hamstring-to-quadriceps ratio (H/Q ratio) (23). Employing the isokinetic centripetal contraction mode at 60 degrees per second, participants executed a series of maximal intensity knee flexion and extension movements. The sequence began with the healthy side and then transitioned to the injured side. The H/Q ratio was recorded for each stage of the test, comprising 5 sets of knee flexion and extension movements following a 5-min warm-up (33). For subsequent group comparison analysis, the average data from the 5 sets of knee flexion and extension were utilized.

### Statistical analysis

For data processing and analysis, this study utilized IBM SPSS 26.0 statistics software (IBM Corp, Armonk, NY, USA). Calculations were presented as mean ± SD, and the normality of data was evaluated using the Shapiro–Wilk test. The main focus of this study was on the comparison between groups. Therefore, to compare outcomes, we employed an analysis of covariance (ANCOVA), which used the baseline outcome data as an adjustment. Count datasets were subjected to comparison using the χ² test for exact probability. Significance was deemed at a threshold of *p* < 0.05.

## RESULTS

### Participants

**Fig. 2:**
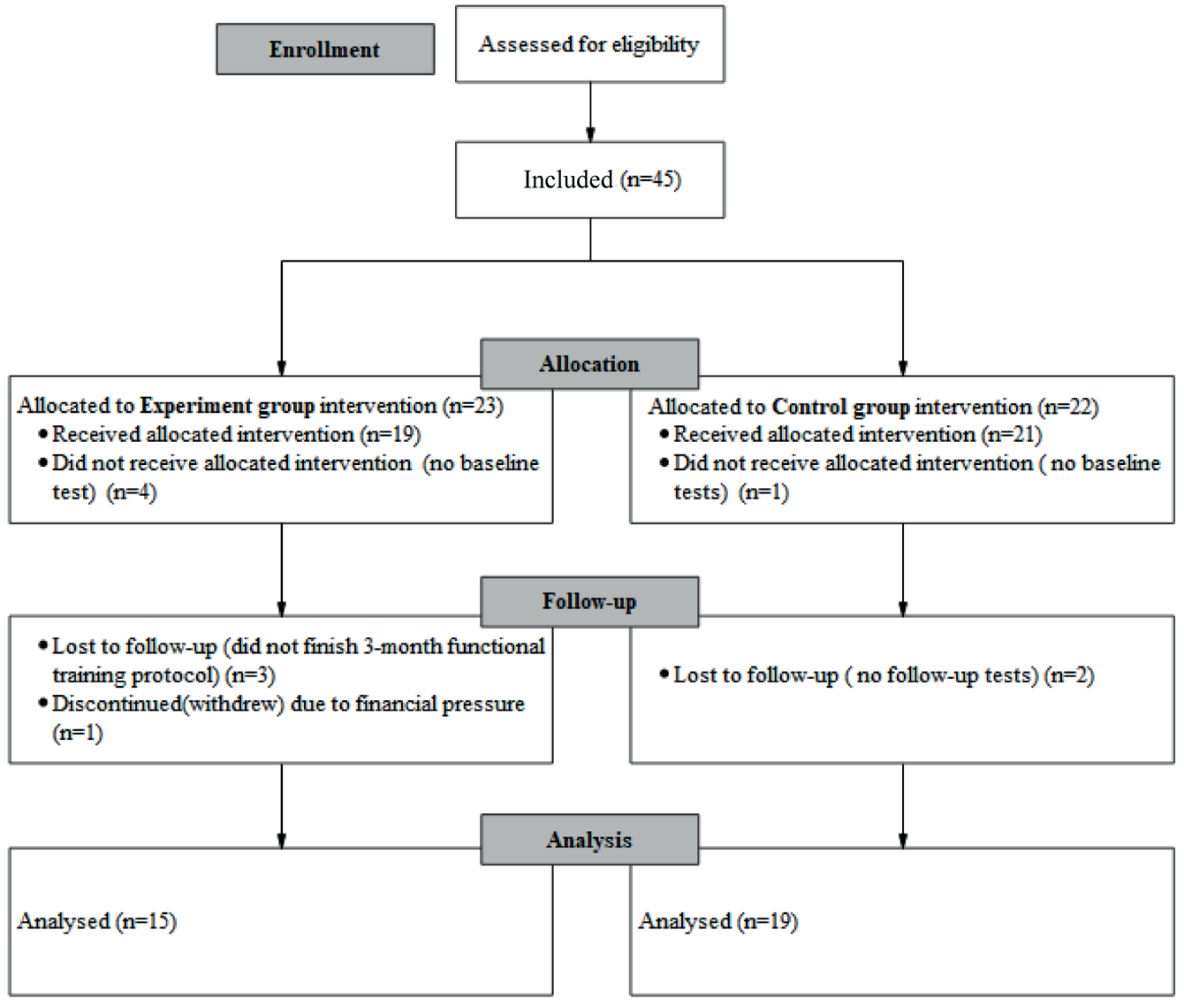
Consort flow diagram.

A total of 34 participants completed the study, divided into 15 in the EG and 19 in the CG (Fig. 2). The EG comprised 6 males (40%) and 9 females (60%), with an average age of 18.8 ± 3.5 years, spanning from 14 to 24 years. The CG included 8 males (42.11%) and 11 females (57.89%), with an average age of 18.5 ± 3.0 years, ranging from 15 to 24 years. Regarding the injured knee, the EG had 8 left (53.33%) and 7 (46.67%), while the CG had 11 left (57.89%) and 8 right (42.11%). Preoperative assessments including the anterior drawer test, joint space tenderness, pivot shift, and Lachman tests were positive for all participants. The measurements including knee range of extension, knee range of extension, Beighton score, and Tegner score showed no significant differences between the 2 groups (all *p*>0.05), as detailed in Table II.

**Table II:** Characteristics of the patients

Variables	Experiment group	Control group	*p*-value
Mean age (y)	18.80 ± 3.50	18.50 ± 3.00	0.809
Gender (%)			0.901
Male	6 (40)	8 (42.11)	
Female	9 (60)	11 (57.89)	
Injured side (%)			0.790
Left	8 (53.33)	11 (57.89)	
Right	7 (46.67)	8 (42.11)	
Knee range of extension	8.55 ± 3.07	8.86 ± 1.76	0.726
Knee range of flexion	22.57 ± 2.99	22.52 ± 3.72	0.965
Beighton score	2.70 ± 1.13	2.53 ± 0.90	0.622
Tegner score	3.23 ± 1.02	3.26 ± 1.05	0.934

### Y-Balance Test (YBT)

The ANCOVA, adjusted for pre-training measurements, revealed significant improvements in both groups post-rehabilitation. The EG demonstrated a 5.25% greater increase in scores for the injured leg and a 6.38% higher increase for the non-injured leg compared with the CG. These results emphasize the efficacy of the functional training in enhancing balance and proprioception in the EG, as shown in Table III.

**Table III:** Comparison of outcomes

Factor	Experiment group (*n* = 15)		Control group (*n* = 19)	*p*-value^#^
Injured YBT				0.014
Pre-	84.31 ± 6.53		81.65 ± 7.25	
Post-	97.24 ± 4.77*		91.99 ± 5.96*	
Healthy YBT				0.961
Pre-	93.93 ± 4.43		87.19 ± 6.42	
Post-	101.0 ± 4.93*		94.69 ± 6.51*	
Injured_IKT				< 0.001
Pre-	36.46 ± 6.35		34.94 ± 5.14	
Post-	67.46 ± 7.31*		56.52 ± 4.56*	
Healthy_IKT				< 0.001
Pre-	74.8 ± 7.10		75.47 ± 7.71	
Post-	73.26 ± 6.11		63.36 ± 3.23	
FMS				
Deep squat				0.001
Pre-	0.53 ± 0.51		0.58 ± 0.60	
Post-	2.07 ± 0.45*		1.58 ± 0.50*	
Hurdle step				0.001
Pre-	1.73 ± 0.45		1.68 ± 0.47	
Post-	2.40 ± 0.50*		1.84 ± 0.37	
In-line lunge				0.001
Pre-	0.73 ± 0.79		0.79 ± 0.78	
Post-	2.27 ± 0.45*		1.74 ± 0.45*	
Shoulder mobile				0.899
Pre-	2.8 ± 0.41		2.74 ± 0.45	
Post-	2.87 ± 0.35		2.84 ± 0.37	
Active straight leg raise				0.827
Pre-	1.47 ± 0.51		1.53 ± 0.51	
Post-	2.53 ± 0.51		2.53 ± 0.51	
Trunk stability push-up				0.831
Pre-	1.87 ± 0.63		1.79 ± 0.63	
Post-	2.00 ± 0.65*		1.95 ± 0.62*	
Rotary stability				0.388
Pre-	1.33 ± 0.81		1.32 ± 0.82	
Post-	1.93 ± 0.25*		1.84 ± 0.37*	

# : Result of analysis of covariance;

* : statistical difference compared with pre-.

### Functional Movement Screen (FMS)

Post-rehabilitation FMS scores indicated significant improvements in the EG, with their scores rising from an initial average of 11.53 ± 1.41 to 16.07 ± 1.22, compared with the CG’s increase from 11.37 ± 1.61 to 14.32 ± 1.53. This demonstrates a substantial reduction in injury risk and improvement in functional movements such as the deep squat, hurdle step, in-line lunge, active straight leg raises, and rotary stability in the EG. No significant differences were noted in shoulder mobility and trunk stability push-up between the groups. These findings are detailed in Table III.

### Isokinetic Knee Testing (IKT)

The post-rehabilitation analysis indicated significant improvements in hamstring/quadriceps (H/Q) ratios in both groups, with notably higher improvements in the EG (*p* < 0.001). Initially, there were significant differences between the injured and healthy sides in both groups. Post-training, the EG showed balanced recovery between the injured and healthy sides, whereas the CG showed a significant decrease in the H/Q ratio on the healthy side, suggesting potential imbalances or compensatory mechanisms. These results are supported by data in Table III.

## DISCUSSION

Numerous rehabilitation regimens investigated in previous studies have sought to enhance recovery outcomes following ACLR, yet their effects have yielded conflicting results. Both ERAS protocols and exercise training have shown potential in mitigating postoperative pain, complications, and accelerating knee functional recuperation (20). Consequently, the present study embarked on a 3-month functional training programme to examine the recuperation trajectory post-ACLR, and ascertain the efficacy of this intensive perioperative rehabilitation regimen. The outcomes unequivocally showcased that individuals undergoing ACLR, in tandem with the ERAS protocol and the 3-month functional training scheme, exhibited notable improvements in various domains encompassing muscle strength and stability of the injured knee, comprehensive dynamic balance, and functional motor performance.

The ACL’s dual role in providing proprioceptive feedback and restraining knee mobility highlights the importance of proprioceptive recovery in rehabilitation (34, 35). The revival of proprioception stands as a paramount early-stage rehabilitation objective. This imperative emanates from its profound correlation with the advancement of joint position sense (36). A swift resurgence of proprioceptive acuity, akin to pre-injury levels, conjoined with accelerated progress in muscle strength recuperation, potentially expedites the patient’s reintegration into sports activities. Scholarly discourse maintains that balance training, particularly when executed in weight-bearing postures, yields improvements in patients’ proprioceptive acumen and facilitates real-time recognition of bodily segmental positioning by the brain (35). Our training programme, which included balance and weight-bearing exercises, contributed to enhanced proprioceptive acuity and muscle strength, and YBT scores were used to evaluated the effectiveness.

An early postoperative training approach, focusing on standing-position exercises, was adopted to amplify motion perception and adjust to individual pain tolerance (16). This strategy resulted in substantial progress in dynamic balance ability, as reflected by higher YBT scores in the EG (36). Notably, the standing-position training, a closed kinetic chain exercise involving weightbearing, activated a broader array of joint receptors, Golgi tendon organs, and muscle spindles (35). Thus, discerning from the YBT results, it is apparent that the functional training exerted a beneficial stimulus towards proprioceptive recovery. In addition to proprioception recovery, a pivotal aspect of the rehabilitation regimen involves incorporating ROM exercises to promote flexion of the injured knee subsequent to ACLR. This imperative has been substantiated through an array of studies. A case in point is the work by Lee et al., where ROM expansion was established as a focal rehabilitation objective for the second week post-surgery. Techniques such as the wall slide and straight leg raises in multiple directions, coupled with cuff and patellar mobilization, were employed to attain this objective (16). Distinct research groups have initiated training immediately following surgery, with the primary aim of enhancing knee mobility. This proactive training approach is believed to forestall quadriceps inhibition, foster cartilage homeostasis, and mitigate potential patellofemoral complications like arthrofibrosis (37, 38). Aligning with Shaw’s approach, the present study encompassed isometric quadriceps training during the initial recovery phase (39). Research by Shaw et al elucidated that isometric quadriceps exercises and straight leg raises could be judiciously incorporated during the initial 2 weeks post-operation. It is posited that such assertive training paradigms confer benefits by expediting knee ROM and bolstering stability in a more rapid manner (40). Our rehabilitation protocol focused on enhancing proprioception and muscle strength of the injured knee, building on the initial gains in proprioceptive enhancement and ROM expansion. Strengthening the hamstring and quadriceps muscles was crucial, as they play key roles in knee joint stabilization and function (6, 9, 41, 42). Our programme included basic training for daily tasks and strength training, emphasizing exercises like bodyweight squats and static lunges to re-establish knee muscle function (15).

The observed outcomes of both the FMS and IKT substantiated the study’s prognostications. The IKT’s H/Q ratio, a widely used parameter delineating knee joint muscle strength properties, gauges the equilibrium between flexor and extensor muscle strength. An optimal H/Q ratio for balanced knee muscles typically hovers around 60% when the knee joint is operating at an angular velocity of 60°/s (43). The attainment of an H/Q ratio above 60% post-ACLR surgery is instrumental in mitigating re-injury risks, as a higher H/Q ratio has been associated with reduced hamstring and ACL injury risks. Conversely, a H/Q ratio below 60% denotes an imbalance in hamstring and quadriceps muscle strength, indicative of knee joint instability (44). In the present study, the H/Q ratio for the injured sides within the EG notably ascended from 36.47% to 67.47% following the 3-month functional training, signifying a significant advancement. Conversely, the CG witnessed a comparatively smaller increment, rising from 36.47% to 56.53%. Furthermore, notable disparities were identified in H/Q ratios between the injured and healthy sides in both EG and CG during the pre-test phase, thus elucidating an initial strength imbalance between the afflicted and unaffected knees. Since all patients in this study underwent ACLR with autologous hamstring grafts, hamstring muscle integrity was inherently compromised due to the surgery itself. Notably, after the 3-month training period, no significant imbalances were noted in H/Q ratios between the injured and healthy sides within the EG. However, modest discrepancies persisted. The H/Q ratio primarily hinges on peak flexion torque, predominantly influenced by hamstring muscle strength. The outcomes underscore that ERAS functional training yields substantial enhancements in postoperative muscle strength of the injured knee joint for ACLR patients, particularly in the realm of hamstring muscles. Conversely, the efficacy of the 3-month spontaneous recovery approach remained relatively inconspicuous. This assertion aligns with conclusions of the study by Setuain et al. (45), underscoring the pivotal role played by the comprehensive functional training protocol, commencing from the initial day following ACLR. This approach significantly contributed to augmenting both hamstring and quadriceps strength, thereby augmenting peak torque and fostering enhanced stability in the knee joint.

The findings derived from the FMS assessments confer heightened credibility to the training’s efficacy. FMS is considered a comprehensive evaluation methodology, serving to appraise functional limitations, muscular imbalances, and asymmetries within individuals (30, 31). Some investigations have leveraged FMS as a predictive tool for athletes’ susceptibility to ACL injuries (46, 47), as well as to gauge the postoperative rehabilitation progress following ACLR surgery (48). It has been suggested that athletes registering FMS scores below 14 points are more predisposed to non-contact ACL injuries compared with those who exceed this threshold (46, 47). Within our study, the juxtaposition of pre- and post-test FMS outcomes between the EG and CG revealed noteworthy insights. The EG’s FMS scores, which surged to 16.07 from 11.53 in pre-tests, surpassed the 14-point threshold (46, 47). This improvement in the EG substantially outstripped the progress within the CG. The evaluations encompassing the deep squat, hurdle step, in-line lunge, active straight leg raise, and rotary stability tests in the EG witnessed conspicuous score enhancements when compared with their CG counterparts (*p* < 0.05). From these outcomes, it can reasonably be deduced that the enhanced functional movement performance attained through EG’s functional training regimen has effectively diminished injury susceptibility. However, the scores from tests pertaining to shoulder mobility and trunk stability push-ups exhibited no statistically significant improvements (*p*>0.05). This underscores that significant impacts were discerned primarily in the realm of lower limb functions, marked by heightened engagement of the knee joints, rather than the upper limbs or trunk. This could potentially be attributed to the training programme’s focused emphasis on augmenting lower limb strength and functionality. Furthermore, the programme’s concentrated effort on facilitating the recovery of injured knees to their pre-injury levels could account for the observed results. This discernible rehabilitation trajectory is reminiscent of the outcomes reported in Przybylak's study, wherein a 12-month supervised physical intervention for ACLR patients culminated in an elevation of FMS scores from 14 to 18 (49). Notably, the present study’s postoperative functional training spanned a mere 3 months, a duration significantly shorter than Lee’s regimen. Despite this temporal disparity, the FMS score improvements in our study marginally exceeded those observed in Lee’s research, albeit with scores in both the pre-test and post-test stages, before and after the 3-month training, registering below their respective benchmarks. This intriguing parallel underscores the comparably efficacious rehabilitative effects across both programmes.

### Limitations

Our study does have limitations. The absence of long-term follow-up data means we cannot fully assess the sustainability of the functional gains. The self-directed rehabilitation in the CG was not monitored, and some preoperative assessment results were not collected. Additionally, the generalizability of our findings may be limited by the specific population of athletes studied and the structured nature of our rehabilitation programme. Future research could benefit from a larger, more diverse sample and extended follow-up to better understand the long-term effects of such training protocols.

### Conclusions

This study sought to enhance the quality of post-surgery recovery using an ERAS approach, which involved a 3-month regimen of functional training. The study aimed to assess the impact of this approach on the knee joint functionality of athletes who had undergone ACLR surgery. The intensive yet methodical training regimen spanning 3 months has significantly contributed to the advancement of proprioception recovery, neuromuscular control, and knee mobility during the early postoperative phase. Furthermore, it has been demonstrated to yield enhancements in muscle strength and function within the lower limbs during the intermediate and later stages of rehabilitation. These findings thus propose that the amalgamation of ERAS principles with the 3-month functional training scheme represents an efficacious programme capable of ameliorating the rehabilitation trajectory. Consequently, this improvement in the rehabilitation process holds the potential to expedite athletes’ return to sporting activities ahead of the natural rehabilitation timeline.
